# Retraction: Enhancing the power quality in radial electrical systems using optimal sizing and selective allocation of distributed generations

**DOI:** 10.1371/journal.pone.0351604

**Published:** 2026-06-15

**Authors:** The PLOS One Editors

The *PLOS One* Editors retract this article [1] due to concerns about compromised peer review.

BB, DKK, K, AAK, YF, and MA did not agree with the retraction. SS responded but expressed neither agreement nor disagreement with the editorial decision. DG and NN either did not respond directly or could not be reached.
